# Polyethyleneimine facilitates the growth and electrophysiological characterization of iPSC-derived motor neurons

**DOI:** 10.1038/s41598-024-77710-1

**Published:** 2024-10-30

**Authors:** Meimei Yang, Daofeng You, Gang Liu, Yin Lu, Guangming Yang, Timothy O’Brien, David C. Henshall, Orla Hardiman, Li Cai, Min Liu, Sanbing Shen

**Affiliations:** 1 Key Laboratory of Measurement and Evaluation in Exercise Bioinformation of Hebei Province, School of Physical Education, Hebei Normal University, Shijiazhuang, 050024 China; 2 Regenerative Medicine Institute, School of Medicine, University of Galway, Galway, H91 W2TY Ireland; 3 FutureNeuro SFI Research Centre for Chronic and Rare Neurological Diseases and Department of Physiology and Medical Physics, RCSI University of Medicine and Health Sciences, Dublin, D02 YN77 Ireland; 4 Emergency Department, The First Hospital of Hebei Medical University, No. 89, Donggang Road, Shijiazhuang, China; 5 Department of Cardiology, Hebei Key Laboratory of Cardiac Injury Repair Mechanism Study; Hebei Key Laboratory of Heart and Metabolism, Hebei Engineering Research Center of Intelligent Medical Clinical Application, Hebei International Joint Research Center for Structural Heart Disease, The First Hospital of Hebei Medical University, Shijiazhuang, China; 6 College of Pharmacy, Jiangsu Key Laboratory for Pharmacology and Safety Evaluation of Chinese Materia Medica, Jiangsu Collaborative Innovation Center of Traditional Chinese Medicine (TCM) Prevention and Treatment of Tumor, Nanjing University of Chinese Medicine, Nanjing, Jiangsu 210023 China; 7 College of Pharmacy, Nanjing University of Chinese Medicine, Nanjing, 210023 Jiangsu China; 8 Confucius Institute of Chinese and Regenerative Medicine, University of Galway, Galway, H91 W2TY Ireland; 9 Department of Physiology and Medical Physics, RCSI University of Medicine & Health Sciences, Dublin, D02 YN77 Ireland; 10 Academic Unit of Neurology, Trinity Biomedical Sciences Institute, Trinity College Dublin, 152-160 Pearse Street, Dublin 2, Ireland; 11 Department of Ophthalmology, Shenzhen University General Hospital, Xueyuan Road 1098, Shenzhen, 518000 China; 12 Ministry of Education Key Laboratory of Molecular and Cellular Biology, Hebei Key Laboratory of Molecular and Cellular Biology, College of Life Sciences, Hebei Normal University, Shijiazhuang, 050024 China

**Keywords:** iPSC-derived motor neurons, Extracellular matrix, Poly-l-ornithine, Matrigel, Polyethyleneimine, Multielectrode array, Biological techniques, Neuroscience, Stem cells, Diseases

## Abstract

Induced pluripotent stem cell (iPSC) technology, in combination with electrophysiological characterization via multielectrode array (MEA), has facilitated the utilization of iPSC-derived motor neurons (iPSC-MNs) as highly valuable models for underpinning pathogenic mechanisms and developing novel therapeutic interventions for motor neuron diseases (MNDs). However, the challenge of MN adherence to the MEA plate and the heterogeneity presented in iPSC-derived cultures raise concerns about the reproducibility of the findings obtained from these cellular models. We discovered that one novel factor modulating the electrophysiological activity of iPSC-MNs is the extracellular matrix (ECM) used in the coating to support in vitro growth, differentiation and maturation of iPSC-MNs. The current study showed that two coating conditions, namely, Poly-L-ornithine/Matrigel (POM) and Polyethyleneimine (PEI) strongly promoted attachment of iPSC-MNs on MEA culture dishes compared to three other coating conditions, and both facilitated the maturation of iPSC-MNs as characterized by the detection of extensive electrophysiological activities from the MEA plates. POM coating accelerated the maturation of the iPSC-MNs for up to 5 weeks, which suits modeling of neurodevelopmental disorders. However, the application of PEI resulted in more even distribution of the MNs on the culture dish and reduced variability of electrophysiological signals from the iPSC-MNs in 7-week cultures, which permitted the detection of enhanced excitability in iPSC-MNs from patients with amyotrophic lateral sclerosis (ALS). This study provides a comprehensive comparison of five coating conditions and offers POM and PEI as favorable coatings for in vitro modeling of neurodevelopmental and neurodegenerative disorders, respectively.

**Supplementary Information:**

The online version contains supplementary material available at 10.1038/s41598-024-77710-1.

## Introduction

Motor neuron diseases (MNDs) are a group of incurable diseases including amyotrophic lateral sclerosis (ALS) and spinal muscular atrophy, that are characterized by progressive degeneration of motor neurons (MNs) located in the central nervous system. MND research has been hindered for a long time due to the inaccessibililty to patients’ MNs. Induced pluripotent stem cell (iPSC) technology and human iPSC-MNs from patients provide an opportunity for developing human in vitro models of MNDs^1^.

However. the variability and reproducibility of experimental findings present significant challenges in utilizing iPSCs^2–5^. The variations may result from the heterogeneity of iPSC clones, and this may be minimized by standardization of iPSC clones, the use of a statistically feasible number of iPSC clones from multiple donors, and the establishment of isogenic iPSC lines if genetic conditions are known^6^. Remarkably, even employing identical cell lines and methodologies, there is a notable variation in experimental outcome across different laboratories^7^. By following the same culture protocol, including handling procedure, culture medium, passage method, coating matrix, differentiation method and others, on would hope to minimize the intra-laboratory or intra-individual variance. This is crucial to ensure the reproducibility of a manifested phenotype of iPSC-drived cells, including iPSC-MNs for underlying pathology of MN degeneration.

Adherence to the culture dish is fundamental to neuronal culture and functional characterization. Extracellular matrix (ECM) is widely used as a coating matrix for culture dishes to support neuronal culture in vitro^8–12^ and plays a crucial role in multiple neuronal functions, such as cell adhesion, proliferation, migration, differentiation, maturation and communication^13^. Over the past decades, there has been significant progress in the field of artificial ECM materials. One notable advancement is the utilization of ECM-mimicking substrates, such as Matrigel or Geltrex, which have facilitated reliable and scalable in vitro expansion and differentiation of iPSCs across laboratories worldwide. However, this has not been successfully extended to long-term culture of MNs^13,14^. Additionally, Matrigel and Geltrex are derived from tumour and composed of undefined ECM components and growth factors, making them potentially xenogeneic.

Scientists have explored synthetic ploycatinoic peptides in combination with chemically defined ECM components, such as, poly-l-ornithine/laminin^15–18^, poly-l-ornithine/laminin/fibronectin^10,19^, and laminin/COLI/COLIV/fibronectin^20^ for in vitro culture of MNs. The use of these defined mixtures of substrates is beneficial for MN culture. However, it is notable that they do not prevent the formation of cell aggregates during neuronal differentiation, as protein- or peptide-based substrates are susceptible to degradation by enzymes that are secreted by the cultured cells^13,21^. Furthermore, purification of ECM components might present challenges due to their complex nature, and variations may also exist in quality control across different batches. The cost associated with these proteins can also be significant.

To address this particular circumstance, scientists have started to develop non-peptide polymer substrates, that are resistant to cellular enzymatic degradation, such as, cytocompatible polypyrrole^22^, polyethyleneimine (PEI)^23^, polypropyleneimine^24^, poly-allylguanidine^25^ and polyelectrolyte multilayers^26^. The synthetic polymers can be utilized either alone or in combination with peptide-based substrates to reach an optimal balance between resistance to protein breakdown and the absence of inherent biological activity. Among the synthetic polymers, PEI has recently been used for in vitro neuronal studies^23^, but not for MNs, especially in coating microelectrode array (MEA) plates^27–31^.

MEA is a valuable platform used to study the electrophysiology of electrogenic cells, such as neurons or cardiomyocytes. It consists of dozens to hundreds of planar electrodes embedded at the base of a culture dish. MEA records extracellular potential changes in electrogenic cells, enabling non-invasive, repetitive and long-term monitoring of electriphysisological activities, particularly neuronal network activities. This platform allows a labor-saving and relatively high-throughput manner to investigate electrophysiological activities. To accurately record electrical signals emitted by neurons, neurons are usually seeded at a very high density ranging from 30,000 to 120,000 cells per mm^2^ on MEA plates^32–34^, to ensure a full coverage of the microelectrodes by the cells.

However, it is well recognized that neurons grown in vitro have a tendency to aggregate into clusters as they mature^16,33–35^. This phenomenon is particularly pronounced when neurons are seeded at high density which aggravates cell attachment. Therefore, when neurons are cultured on MEA plates, notable cell detachment and loss of cultures may occur, and this interfers with the precise recording of genuine neuronal electrical signals^16,33,34^. Thus, it is crucial to identify a coating matrix that enables an even distribution and firm attachment of neurons to attain reliable electrophysiological recording of true neuronal signals from the MEA plates.

PEI presents a high density of cationic charges under the physiological pH conditions. Yet, unlike poly-lysine and poly-ornithine, PEI does not contain peptide bonds, making it remarkably resistant to proteolysis^23,31,36^. In comparison to other commonly used coating substrates, such as poly-lysine, laminin, poly-l-ornithine and fibronectin, PEI alone or in conjunction with laminin was shown to result in enhanced cell adherence to the culture dish and a more homogeneous distribution of neurons^23,36^. However, untill now, there have been no reports on its use for the MNs or its potential impact on electrophysiological properties in culture.

In this study, we compared cell attachment and electrophysiology of iPSC-MNs among five coating conditions, and both PEI and POM (poly-l-ornithine/Matrigel) were found to support the differentiation and maturation of MNs. Furthermore, both PEI and POM coatings can be used to detect aberrant electrophysiological phenotypes of iPSC-MNs from individuals with sporadic ALS (sALS) using MEA technology. While considering the cost, simplicity, reproducibility and stability of investigation into the pathophysiology of MNs or neurons from MND patients, we recommend PEI, which allows continuous evaluation of spontaneous network activities of neurons as they mature and ‘aged’ in an in vitro environment for up to seven weeks.

## Results

### CHAT^+^ spinal MNs are derived from iPSCs in 28 days by following a published protocol

**Fig. 1:**
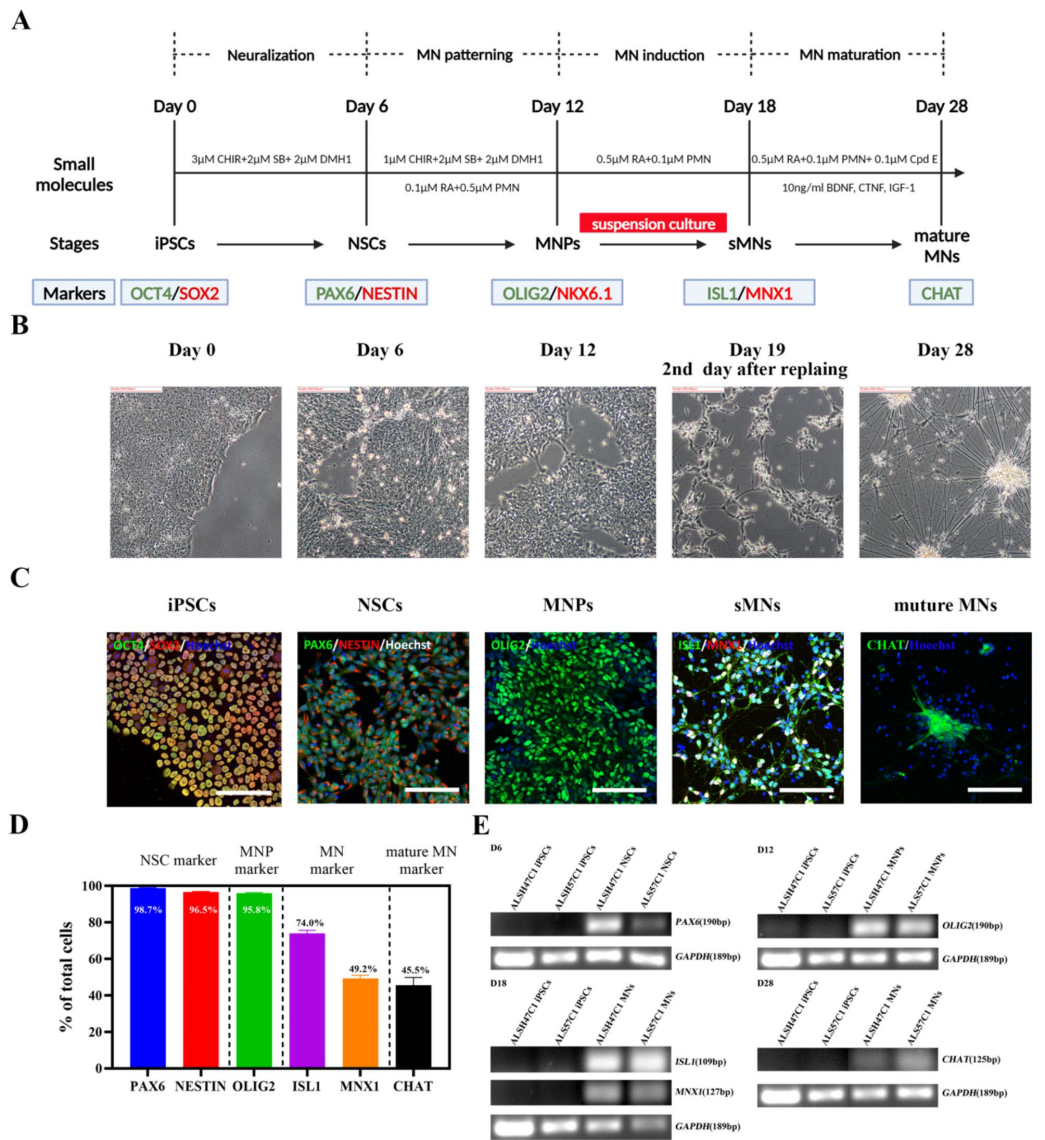
CHAT^+^ spinal MNs can be induced from iPSCs in 28 days using a previously published protocol. (**A**) Schematic diagram of the 28-day MN differentiation protocol established by Du et al^8^. **(B)** Representative morphology of cells during the course of differentiation from day 0 to day 28. **(C)** Representative staining of cells at each differentiation stage with stage-specific markers. OCT4/SOX2 staining for iPSCs on day 0, PAX6 and NESTIN staining for NSCs on day 6, OLIG2 staining for MNPs on day 12, ISL1 and MNX1 staining for early MNs on day 18 and CHAT staining for mature MNs on day 28 of differentiation. **(D)** The proportion of PAX6^+^ and NESTIN^+^ cells on day 6, OLIG2^+^ cells on day 12, ISL1^+^ and MNX1^+^ cells on day 18 and CHAT^+^ cells on day 28 of differentiation. N = 3 cell lines, n = 3 replicates. All representative images were from the iPSC line ALSH47C1. Scale bar, 100 µm. See also Fig. S1. **(E)** RT-PCR data showing the mRNA expression of *PAX6* on day 6 (left up), *OLIG2* on day 12 (right up), *ISL1* and *MNX1* on day 18 (left bottom) and *CHAT* on day 28 (right bottom) of differentiation compared to iPSCs on day 0. *GAPDH* was used as a house-keeping reference gene. Full-length gels are presented in Fig. S2.

The iPSCs were first differentiated into spinal MNs using a previously established protocol^8^. The differentiation process was artificially divided into four stages: iPSC neuralization, MN patterning (ventralization and caudalization), MN induction and MN maturation (Fig. 1A). The morphology of differentiated cells was monitored throughout the differentiation until day 28 (Fig. 1B). To induce MNs, cells were adherently cultured for 12 days and then in suspension (Figure S1) for another 6 days. On day 18, the cells were dissociated into single cells, replated onto poly-l-ornithine/laminin (POL)-coated plates, and treated with 0.1 µM compound E to promote the differentiation and maturation of spinal MNs (Fig. 1A). After replating, iPSC-derived cells began to project neurites, displaying neuronal identity. However, cells progressively formed long neurite bindles and cell bodies aggregated into large clusters by day 28 of differentiation (Fig. 1B).

The identity of differentiated cells was verified at each differentiation stage by quantitative RT-PCR and immunocytochemistry for the expression of specific markers (Fig. 1C,D). The iPSCs, confirmed by high expression of the pluripotent marker OCT4/SOX2 (Fig. 1C), were induced to homogenous neural stem cells (NSCs) after 6 days of treatment with the WNT activator CHIR99021, dual-SMAD inhibitors SB431542 and DMH1. Approximately 98.7 ± 0.2% of the cells were positive for the NSC marker of PAX6, 96.5 ± 0.3% of the cells expressed NESTIN (Fig. 1C), whereas OCT4 expression was suppressed (Fig. S1B). Subsequently, the NSCs were directed into MN progenitors (MNPs) after another 6-day treatment of retinoic acid (RA) and purmorphamine (an agonist of SHH) in addition to SMAD inhibition and WNT activation (Fig. 1A), and 95.8 ± 0.5% of the cells expressed the MNP marker of OLIG2 (Fig. 1C,D).

The OLIG2^+^ MNPs were cultured in suspension with RA and purmorphamine for 6 days (Fig. 1A), resulting in the generation of 73.8 ± 2.3% ISL^+^ and 49.3 ± 1.7% MNX1^+^ MNs by day 18 of differentiation (Fig. 1C,D). Subsequently, Compound E was applied to the cultures to promote MN maturation (Fig. 1A). However, only 45.5 ± 4.2% of cells were found to express the mature MN marker of CHAT after a maturation period of 10 days (Fig. 1C,D). The expression of specific markers at each stage was also validated at the mRNA level using RT-PCR analysis (Fig. 1E). The proportion of mature MNs was substantially less than previously reported^8^, which was reproduced in three independent iPSC lines (Fig. S1).

### Optimal adherence and morphology of iPSC-MNs under POLFM and POM coating for over 4 weeks

**Fig. 2:**
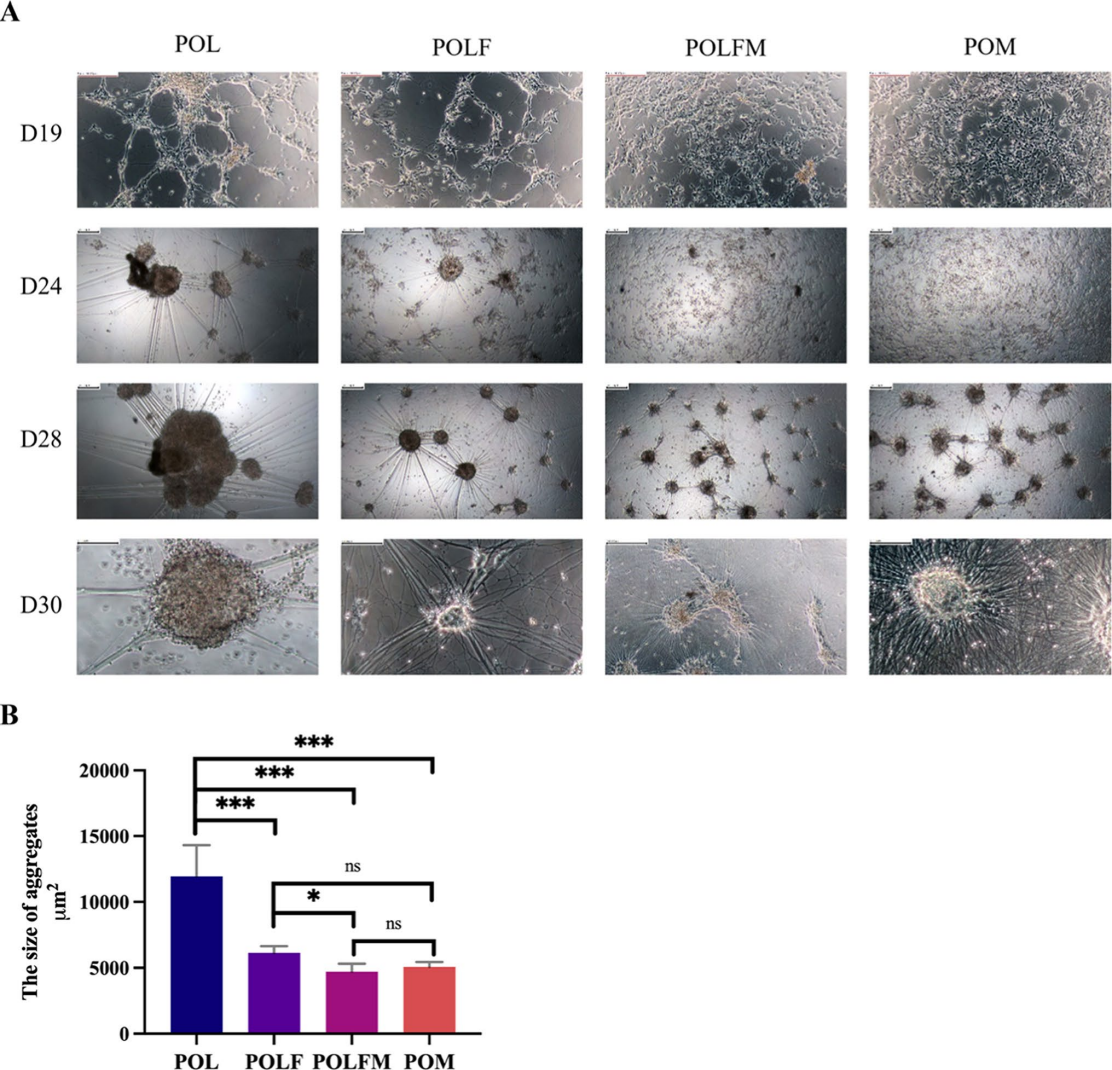
POM and POLFM coating conditions showed improved adherence, neurite development and smaller aggregates of iPSC-MNs. (**A**) Representative morphology of the cells grown on dishes pre-coated with POL, POLF, POLFM or POM (from left to right) and images were taken on day 19, 24, and 28 (from top to bottom). Scale bar 100 μm. **(B**) The quantification of aggregate size among four coatings on day 28 of differentiation. N = 3 biological replicates. The data were presented as mean ± SEM. Variations among groups were examined for statistical significance using unpaired student’s *t* test. *p < 0.05, **p < 0.01, and ***p < 0.001.

MNs are non-adherent cells and require an ECM to support in vitro attachment and growth. Our initial culture experiments showed that the commonly used coating condition of poly-l-ornithine/laminin (POL) coating condition could not support the long-term adherence of iPSC-MNs, and this was also reported previously^15,17,37,38^. The cells showed a tendency to aggregate into large clusters from day 24 of differentiton and often detached from the culture dishes duringmedium change after > day 30 of differentiation prior to downstream analyses (Fig. 2A Lane 1), which was consistant with previous studies^16,30,34^. There were three multiple peptide substrates, namely POLF (POL + laminin + Fibronectin), POLFM (POLF + Matrigel) and POM (Poly-L-ornithine/Matrigel) that were previously employed for MN cultures in the literature (Table 2)^9–12^. We therefore conducted a comparative analysis of four coating conditions (POL, POLF, POLFM and POM) to assess which condition could best promote firm attachment and even distribution of iPSC-MNs in vitro.

By following Du’s protocol, we dissociated suspension cultures (Fig. S1A) into single cells on day 18 of differentiation, and replated onto a 96-well plate, which was pre-coated with four different coating conditions, and closely monitored cell attachment throughout the differentiation (Fig. 2). From day 24 onwards, cells on POL- and POLF-coated wells started to cluster into small aggregates (Fig. 2A, Lanes 1 and 2), whereas cells cultured on POLFM and POM-coated wells continued to display monolayer growth (Fig. 2A, Lanes 3 and 4). During the subsequent culture, the aggregates on POL- and POLF-coatings progressively increased in size became large spheres, resulting in loose contact to the culture dish (Fig. 2A, Lanes 1 and 2). Consequently, the aggregates could easily be detached from the culture dish during medium replenishment (from ~ day 30 of differentiation). Although cell aggregates were also observed on the POLFM or POM coated-wells by day 28 of differentiation (Fig. 2A, Lanes 3 and 4), these aggregates were substantially smaller in size than those grown on POL- and POLF-coated wells and remained flat in morphology (Fig. 2B). In addition, iPSC-MNs exhibited a significant increase in the formation of neurite networks on the POLFM or POM coating conditions, suggesting that neurons developed a higher maturity under these two coating conditions (Fig. 2A, Row 4).

In summary, the cell attachment assay demonstrates that both POM and POLFM can improve adherence and maturation of the iPSC-MNs. Whereas POLFM coating requires Poly-L-ornithine (4℃ overnight), laminin/fibronectin (37 °C, 2 h) and Matrigel (37 °C, 2 h) at a cost of €11.38/ml, the POM coating contains only Poly-L-ornithine and Matrigel, and is more economical (€1.79/ml) (Table 2 and Table S1) and easier to manipulate. The POM coating was therefore deployed in the subsequent electrophysiological research.

### PEI coating promotes firmer attachment and more homogeneous distribution of iPSC-MNs on MEA plates compared to POM coating in 7-week of MN culture

**Fig. 3:**
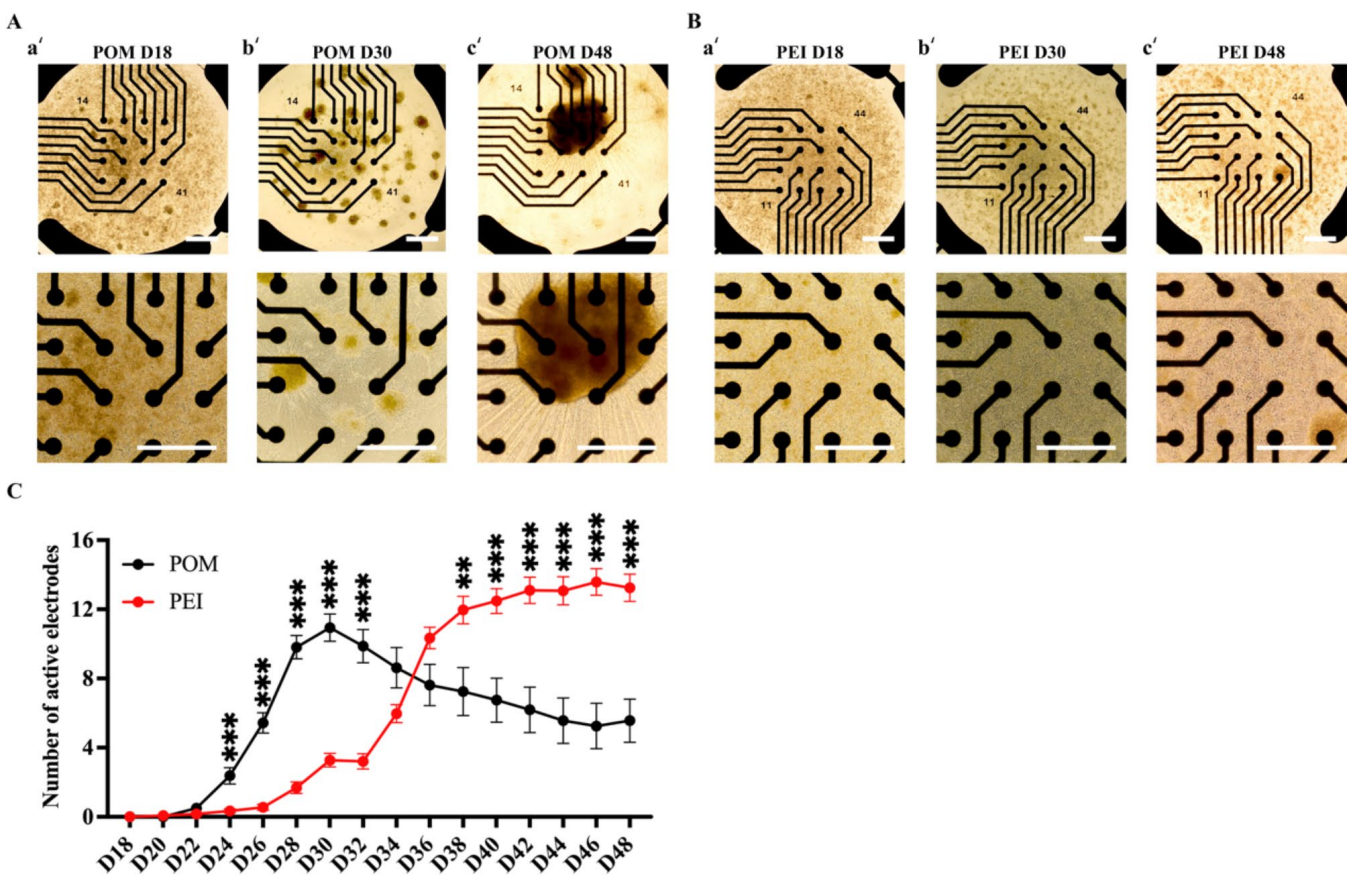
PEI coating condition promoted an even distribution of iPSC-MNs on the MEA plates in an Supplementary culture. **(A-B)** Representative morphology of iPSC-MN cultures on day 18, day 30 and day 48 from 48-well MEA plates coated with POM (A_a’-c’) or PEI (B_a’-c’). The top panels were taken under 4 × magnification, and the bottom panels were taken from the respective well on the top but under 10 × magnification. Scale bar, 100 µM. **(C)** Plots of MEA recording data for the number of active electrodes from the longitudinal recording of day 18 to 48 of iPSC-MNs on the POM or PEI-coating conditions. The active electrodes was defined as ≥ 5 spikes/min. The total recorded wells were n = 24 in the POM-coating condition and n = 45 in the PEI coating condition from three independent cell lines. Data were presented as mean ± SEM. Variations among groups were examined for statistical significance using multiple unpaired student’s *t* test. *p < 0.05, **p < 0.01 and ***p < 0.001. See also Table S2.

MEA plates were subsequently coated with POM for a long-term MN culture, and adherence and functional maturation of iPSC-MNs were investigated by electrophysiological characterization*.* Similar to previous observation on the POM-coated conventional culture dishes, small cell clusters started to appear on the POM-coated MEA plates from day 30. However, the small aggregates kept growing in the subsequent 18 days, and large MN aggregates became evident by day 48 (Fig. 3A). The number of active electrodes (defined as ≥ 5 spikes/min) were quantified, which was found to peak around day 28–30 on the POM-coated dishes but declined during the subsequent culture (Fig. 3B), which was co-related with the increasing growth of MN aggregates and worsening cell attachment (Fig. 3A_a’-c’).

In searching for more optimal coating substrates for MN aging on the MEA plates, a non-peptide polymer, PEI, was found employed in an early study for neuronal culture due to its ability to enhance neuronal adhesion and to minimize cell aggregation on culture dishes on MEA plates^23^. However, there has been no report documenting its utilization for in vitro MN culture. We therefore, tested PEI in this study and compared with POM for MN growth on the MEA plates.

In addition, we found that the sMN differentiation efficiency was substantially lower in our experiments than that previously reported^8^. We therefore developed a novel monolayer sMN differentiation protocol recently to improve the MN production^39^. The addition of a NOTCH pathway inhibitor, Compound E, was advanced to day 12 (instead of day 18) for a duration of 6 days, and this timely enabled conversion of MNPs to MNs, and resulted in an efficient generation of 91.2 ± 7.0% of MNs expressing CHAT^39^.

We therefore switched to this monolayer differentiation protocol to generate MNs for the subsequent electrophysiological investigation. MNPs were dissociated on day 12 and reseeded at a cell density of 50,000 cells/well onto the POM or PEI-coated 48-well MEA plates. Neuronal attachment on the MEA plates was closely monitored throughout the culture process (Fig. 3). As anticipated, MNs on POM-coated MEA plates displayed small clusters by day 30 of differentiation, and large aggregates appeared by day 48 (Fig. 3A). On the other hand, the MNs cultured on the PEI-coated wells did not display obvious cell clusters on day 30, and no appearance of large aggregates by day 48 (Fig. 3B). Accordingly, the number of active electrodes on the PEI-coating was gradually increased and stabilized around day 40 to day 48 (Fig. 3C), which was benefited from even distribution of MNs. Taken together, the PEI coating was shown to be more suitable to enhance firm adhesion in a long-term and a more homogeneous distribution of iPSC-MNs than POM. No major cell clustering issue occurred over 36 days of MN culture on the MEA plate, even with a high seeding density of 50,000 cells on a 1.1 × 1.1 mm recording area of 48-well MEA plate, which showed its suitability for MND research.

### Electrophysiological signals of iPSC-MNs are influenced by coating conditions

**Fig. 4:**
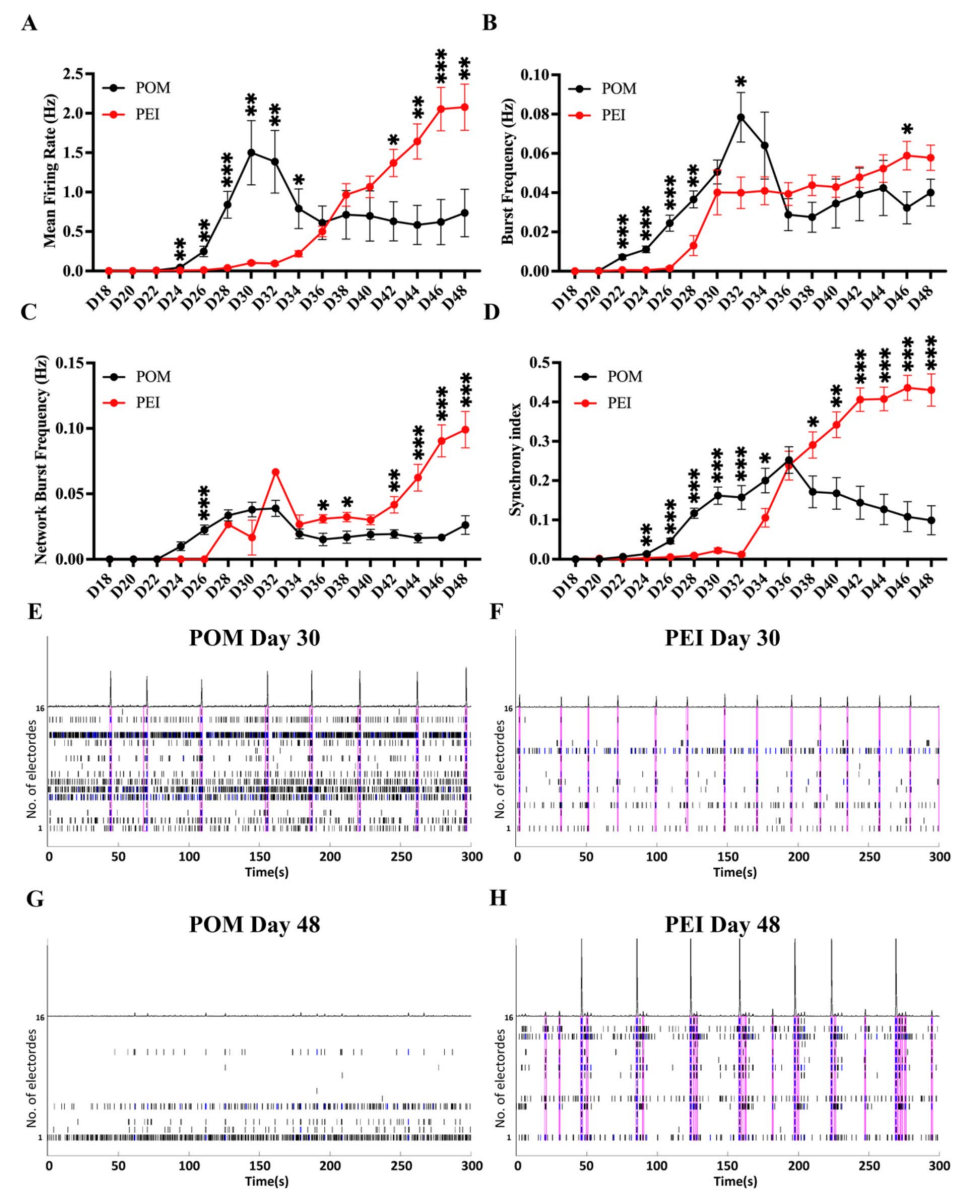
The iPSC-MNs showed different electrophysiological characteristics under the POM and PEI coating conditions. **(A-D)** Plots of MEA recording data for the mean firing rate (Hz) **(A)**, burst frequency (Hz) **(B),** network burst frequency **(C)** and synchrony index **(D)** from the longitudinal recording of day 18 to 48 of iPSC-MNs on the POM or PEI-coating conditions. **(E–H)** Roaster plot of MEA recordings showing representative changes in spike firing pattern on day 30 (**E**, **F**) or 48 (**G** and** H**) on the POM- (**E** and** G**) or PEI- (**F** and** H**) coated 48-well MEA plates. The ticks indicate the time of a neuronal action potential or “spike” detected, and each row indicates firing of one electrode. The series of blue ticks indicate the bursts detected in 100 ms ISI. The ticks included in a network burst were outlined by magenta boxes. Above the raster is a filtered population spike time histogram, showing the total number of spikes occurring throughout the well at each time, and a higher peak represents more spikes detected at that time point, as a synchrony index in some extent. In this study, the total recorded wells were n = 24 in the POM-coating condition and n = 45 in the PEI coating condition from three independent cell lines. The data were presented as the mean ± SEM. Variations among groups were examined using multiple unpaired student’s *t* test. *p < 0.05, **p < 0.01 and ***p < 0.001. See also Figs. S3–S4 and Tables S2–S3.

Electrophysiological characteristics are essential for evaluating the functionality of mature neurons, but little is known about whether they could be influenced by coating conditions. To compare the electrical performance of hiPSC-MNs on the POM- and PEI-coating conditions, the firing properties of the MNs were recorded every other day from day 18 to 48 of differentiation on 48-well MEA apparatus. Distinct firing patterns of spontaneous activities were observed under the two different coating conditions, as the iPSC-MNs progressively matured on the MEA plates (Fig. 4).

In comparison to the PEI-coating, the iPSC-MNs on the POM-coated MEA wells exhibited accelerated development of spontaneous firing activities at the early stage of differentiation (Fig. 4). The mean firing rate (MFR) and burst frequency from the iPSC-MNs on the POM-coating increased sharply from day 22 and peaked at day 30–34 of differentiation, which were significantly greater than those cells grown on the PEI-coated wells (Fig. 4A,B). In addition, the cells on the POM-coated wells showed stronger network activities by day 30 (Fig. 4C,E) and higher synchronization by day 34 (Fig. 4D) in comparison to those grown on the PEI-coated wells, indicating a greater functional connectivity among neurons. After peaking neuronal activities at day 30–34 of differentiation, however, there was a subsequent decline of the electrical activities from day 32 from the POM-coated wells among four parameters measured (Fig. 4A–D and G), which were coincided with progressive formation of large aggregates of MNs during the in vitro “aging” on MEA plates, which also aligned with previous findings on conventional cultureware (Fig. 3).

On the PEI-coated wells, however, a uniform distribution of cells was observed throughout the entire culture duration up to the end of recording by day 48 (Fig. 3B). The iPSC-MNs exhibited a slower development of functional firing at the early stage of differentiation compared to cells on the POM-coating, but a rapid increase of the firing activities appeared from day 34. The electrical activities on the PEI-coated wells continued to rise and were significantly higher than those on the POM-coated wells from day 40 to 48 (Fig. 4A–D, F, H). This firing pattern and data was repeatedly shown among three different iPSC lines (Fig. S4 and Table S2). These findings indicate that different coating conditions can influence the maturation speed of iPSC-MNs in an in vitro setting. Furthermore, coating conditions can also affect the stability of electrical signals detected on the MEA, via influencing MN adherence.

### sALS iPSC-MNs display spontaneous hyperexcitability

Changes in the excitability of MNs were previously reported in both in vivo and in vitro models of ALS, such as in ALS patients^40,41^ and in MNs derived from patients with familial ALS who carried known mutations^35,42,43^, as well as in transgenic animal models of ALS^44–46^. This prompted us to investigate whether these coating conditions could equally impact the electrophysiological properties of iPSC-MNs from sALS patients and controls.

**Fig. 5:**
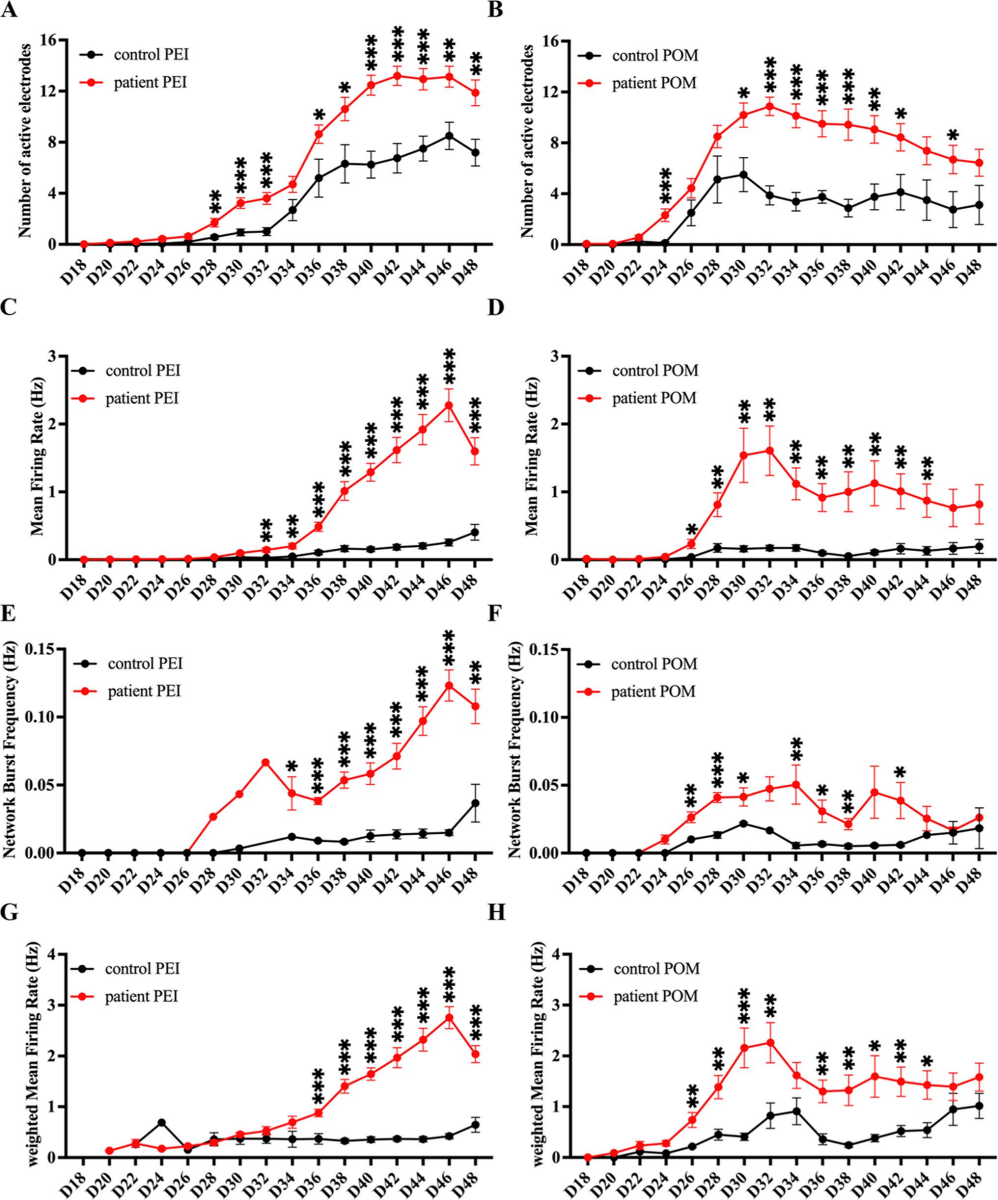
The PEI and POM coating enabled detection of hyperexcitability of iPSC-MNs from sALS patients. Plots of MEA recording data for the number of active electrodes (**A**, **B**), mean firing rate (Hz) (**C**, **D**), network burst frequency (**E**, **F**) and weighted mean firing rate (**G**, **H**) from the longitudinal recording of day 18 to 48 of iPSC-MNs on the PEI or POM-coating conditions. The total recorded wells were n = 45 for the PEI coating condition and n = 24 for the POM-coating condition from three independent cell lines. The data were presented as the mean ± SEM. Variations among groups were examined using multiple unpaired student’s *t* test. *p < 0.05, **p < 0.01 and ***p < 0.001. See also Fig. S4 and Tables S3–S4.

Both groups of MNs were cultured in parallel on the coating substrates of POM or PEI (Fig. 5). The sALS iPSC-MNs consistently displayed an increased excitability under the PEI-coating condition, when compared to the control iPSC-MNs, and this was evidenced by the significantly higher values of the number of active electrodes, MFR and network burst frequency (Fig. 5A,C,E). On the POM-coated wells, sALS iPSC-MNs also showed a similar result from day 26 to 44, but the signals of all parameters started to decrease around day 30–32 and led to no significant difference during day 46 and 48 between sALS and control iPSC-MNs (Fig. 5B,D,F). This discrepancy could be attributed to the formation of neuronal aggregates and subsequent loose contact of the cells on the POM-coated wells (Fig. 3_c’), which led to greater variations compared to cells cultured on the PEI-coated wells.

To minimize the impact of inactivated electrodes resulting from neuron detachment, we compared the weighted MFR (wMFR), which exclusively considered the firing rate of active electrodes at each time point, and discarded those that were inactive. The value of wMFR was found to be higher than that of MFR when inactive electrodes were omitted. However, this did not alter the hyperexcitability phenotype of sALS iPSC-MNs, regardless of the coating conditions used (Fig. 5G,H). The raw data of the individual control and patient groups were included in Fig. S4 and Table S3. The enhanced excitability of sALS iPSC-MNs was consistently reproduced in subsequent experiments using a larger sample scale under the PEI coating, which was recently reported^39^. In conclusion, altered excitability was effectively captured in the sALS iPSC-MNs under the PEI- or POM-coating condition, in terms of consistent firing quantity and enhanced synchronization of neurons. Therefore, PEI is a more suitable choice for investigating electrophysiological activity of cells on the MEA plates, particularly for long-term studies of neurodegenerative disorders.

## Discussion

MEA has been extensively employed in research on cardiac disease and neurodevelopmental/neurodegenerative disorders^28,47^ due to its non-invasiveness, labor-saving and relatively high readouts. These attributes make MEA suitable for investigating disease progression and conducting in vitro drug testing. In combination with iPSC-MN models, MEA enabled recapitulation of abnormal electrophysiological activities from MND patients, which led to the discovery of novel candidate drugs such as retigabine^48^. However, the clustering of iPSC-MNs takes place on MEA plates during the long-term culture in vitro, which not only creates a significant challenge for investigating aging-related phenotypes, but also potentially hinders the reproducibility of pre-symptomatic data and limits screening of novel drugs for the treatment of MNDs.

In this study, a comparative analysis was conducted to examine five coating conditions for their impact on the attachment and electrophysiological performance of iPSC-MNs. The initial step involved comparison of POL, POLF, POLFM and POM on conventional culture ware for up to 30 days of culture, and the iPSC-MNs exhibited better distribution on the surfaces coated with POLFM or POM. Considering that POM coating contains only poly-l-ornithine/Matrigel and lacks expensive laminin and fibronectin, but yields a similar outcome to the POLFM with laminin and fibronectin, the POM coating is recommended for MN culture on conventional or MEA dishes for up to 5 weeks. In addition, POM appeared to accelerate neuronal maturation in the early phase of MN differentiation, strongly and linearly increased neuronal activities were detected in iPSC-MNs from day 24 to 30 (Fig. 3). Therefore, POM coating can be adopted to investigate stem cell models of neurodevelopmental disorders.

However, large aggregates did form in the prolonged culture under the POM-coating condition, which led to reduced contacts with microelectrodes and decreased MEA signals detected during the subsequent 2 weeks of MN culture. This was likely due to the degradation of the peptide matrix of poly-l-ornithine and Matrigel by the enzyme secreted from the cells^13,21^.

In terms of neurodegenerative dieases, it is more desirable to use “aging” cell models. It has been reported that reprogramming iPSCs revert some key hallmarks of cellular age, such as epigenetic age^49,50^. Therefore, a more stable culture condition that can extend the in vitro culture time of iPSC-derived neurons would partially benefit and facilitate the “aging” on culture to recapitulate aging-related phenotypes. Therefore, an optimal ECM, or co-culture with astrocytes or other coating methods were developed to achieve more homogeneous cell distribution in the longer term, which would benefit the utilization of MEA for electrophysiological phenotyping for neurodegenerative diseases^16,34^. In this respect, we compared the POM and PEI coating conditions, and observed that the PEI exhibited greater suitability for MND modeling. There was no significant formation of large aggregates over the course of 7 weeks of MN cultures in the PEI wells, and neuronal electrical activities were consistently increased until the end of 48 days of differentiation. Notably, the cells demonstrated greater synchronization on the PEI-coated wells than that on POM-coated wells. Electrophysiological characteristics of iPSC-MNs showed that the PEI-coating condition enabled the capture of increased spontaneous excitability of MNs derived from sALS iPSC lines, which was consistent with previous findings in familial ALS iPSC-MN models^35,42,51–53^. Therefore, we recommend PEI-coating for neurodegenerative research.

However, it is worth mentioning that there was a delay in detecting electrical signals from the PEI-coated wells during the initial phase, and the strength of the MEA signals was also reduced in comparison to the POM-coated wells. The exact cause of this discrepancy was unknown, and previous studies suggested that the lack of bioactivity and potential toxic effects of PEI might partially contribute to this^54^. Therefore, apoptosis and senescence of the neurons on PEI coating, despite not obvious in the current study, would be measured to address this question. Another limitation is that the PEI coating is limited to 7-weeks of MN culture, as some small aggregates started to form after day 48 and electrical signals started to drop since then (data not shown). Coating conditions for iPSC-MN culture beyond 7 weeks are yet be identified.

Recently, a new non-peptide polymer, known as dendritic polyglycerol, an amine-based substrate, in combination with Matrigel, was reported to significantly improve the long-term culture of iPSC-MNs^21,33^. This enhanced culture system allowed sustained investigation of various aspects, including cell viability, molecular characteristics, spontaneous network electrophysiological activity, and single-cell RNA sequencing of iPSC-derived mature MNs for up to two months. Matrigel, being a composite of ECM components, is deemed suboptimal for in vitro investigations. Laminin, an essential component of Matrigel, has been extensively employed in neuronal research in combination with other synthetic peptide, such as poly-l-lysine, poly-D-lysine and poly-l-ornithine^28^. A potential resolution to accelerate maturation of iPSC-MNs might be achieved using the PEI coating in combination with laminin or other synthetic peptide, which could be examined in a forthcoming investigation.

In summary, this study demonstrated the beneficial effects of the PEI coating for the investigation of iPSC-MNs up to 7 weeks, leading to a more uniform distribution, the feasibility of investigating mature iPSC-MNs and the stable acquisition of electrophysiological activities. Consequently, the improved long-term culture of iPSC-MNs will contribute to the investigation of neurodegenerative diseases and support the early-stage of drug discovery efforts for MND diseases.

## Materials and methods

### Maintenance of iPSCs

The iPSC lines used in this study are listed in Table 1 and were characterized and described previously^55,56^. All iPSC lines were maintained on Geltrex™ (A1413302, Gibco)-coated 6-well plates in Essential 8™ Flex Medium (A2858501, Gibco).

**Table 1:** Information of cell lines used in the study.

Status	Cell line	Sex	Age	Ethnicity
Healthy	NUIGi049-A (ALSH47C1)	66	Female	Caucasian
Sporadic ALS	NUIGi050-A (ALS53C5)	60	Male	Caucasian
Sporadic ALS	NUIGi051-A (ALS57C1)	56	Female	Caucasian

### MN differentiation

MNs were initially differentiated following a previously published protocol with slight modification for comparison of four coating conditions^8^. In this study, iPSCs at passage 20–30 were dissociated with Accutase (A6964, Sigma) and seeded at 30,000 cells/cm^2^ on (1:100) Geltrex (A1413302, Gibco) -coated 6-well plates in Essential 8 flex medium supplemented with 10 μM Y-27632 (72304, STEMCELL) on day -1. The Neuronal Induction Medium (NIM) consisted of 1:1 DMEM/F12 (BE-12-719F, Lonza) and Neurobasal medium (21103049, Gibco), 1% P/S (15140122, Gibco), 0.5 × N2 (17502048, Gibco), 0.5 × B27 (17504044, Gibco), 0.1 mM Ascorbic Acid (A4403, Sigma) and 1% GlutaMAX (35050061, Gibco). On day 0 and every other day for the subsequent 6 days, the cells were replenished with NIM medium and 3 μM CHIR99021 (HY-10182, MCE), 2 μM SB (HY-10431, MCE), and 2 μM DMH1 (HY-12273, MCE) were freshly added. Cells were subsequently split 1:3 onto Geltrex-coated plates in NIM containing 1 μM CHIR, 2 μM SB, 2 μM DMH1, 0.1 μM RA (HY-14649, MCE) and 0.5 μM Purmorphamine (HY-15108, MCE) for the following 6 days, with the medium changed every two days.

On day 12 of differentiation, patterned cells (defined as MNPs) were accutased into single cells and split 1:3 into 6-well plates pretreated with anti-adherence rinsing solution (07,010, Stemcell Technologies) for suspension culture in NIM supplemented with 0.5 µM RA and 0.1 µM Purmorphamine from day 12 until day 18. Then, the suspension cultures were accutased into single cells and replated on culture ware pre-coated with POL, POLF, POLFM or POM in the NIM with 0.5 μM RA, 0.1 μM Purmorphamine, 0.1 μM Compound E (HY-14176, MCE), along with three neurotrophic factors (NTFs) of 10 ng/ml BDNF (450–02, PeproTech), 10 ng/ml CNTF (450–13, PeproTech), and 10 ng/ml IGF-1 (450–10, PeproTech) for 10 days. Half of the medium was gently changed every other day to prevent disruption to the neurons until day 28 of differentiation. From day 28 onwards, RA, purmorphamine and compound E were all removed from the medium until the cells were ready for subsequent analysis.

For electrophysiological comparative analysis, MNs were differentiated using our recently published protocol^39^. In short, after 12 days of differentiation with Du’s protocol^8^, cells were dissociated and replated for monolayer culture onto culture ware pre-coated with POM or PEI in the NIM supplemented with 0.5 μM RA, 0.1 μM Purmorphamine and 0.1 μM Compound E, along with three NTFs to facilitate differentiation and maturation of iPSC-MNs. On day 18 of differentiation, RA, Purmorphamine and Compound E were removed from the medium, and half of the medium was carefully changed every other day until day 48.

### Coating matrix preparation

The detailed information of coating reagents is listed in Table 2.

**Table 2:** ECM coating conditions used in this study.

Abbreviation	ECM components	References	Cost(€)/ml*
POL	20 μg/ml Poly-L-ornithine + 20 μg/ml Laminin	^9^	5.15
POLF	20 μg/ml Poly-L-ornithine + 20 μg/ml Laminin + 10 μg/ml Fibronectin	^10^	8.23
POLFM	20 μg/ml Poly-L-ornithine + 20 μg/ml Laminin + 10 μg/ml Fibronectin + Matrigel (1:20)	^12^	11.38
POM	20 μg/ml Poly-L-ornithine + Matrigel (1:50)	^11^	1.79
PEI	0.1% Polyethyleneimine in borate buffer		0.11

* The detailed calculation is listed in Table S1.

**POL**: 100 µg/ml Poly-L-ornithine (P4957, Sigma) was diluted to 20 µg/ml in Dulbecco’s Phosphate Buffered Saline (DPBS, D8662, Sigma). The culture ware was coated with 20 µg/ml Poly-L-ornithine at 4℃ overnight. Laminin at 1 mg/ml (L2020, Sigma) was thawed at 4℃ overnight and diluted to 20 µg/ml in DPBS. On the second day of coating, Poly-L-ornithine was removed, and the plates were rinsed with sterile water twice and then coated with 20 µg/ml of Laminin at 37 °C for 2 h. The coating matrix was removed and rinsed once with PBS before plating cells.

**POLF**: The dishes were first coasted with poly-l-ornithine as described above. The 1 mg/ml laminin and 1 mg/ml fibronectin (F1141, Sigma) stocks were pre-thawed at 4℃ and diluted with DPBS to contain 20 μg/ml laminin and 10 μg/ml fibronectin. Then poly-l-ornithine pre-coated plates were coated with laminin/fibronectin solution at 37 °C for 2 h. The coating matrix was removed and rinsed once with PBS before plating cells.

**POLFM:** After poly-l-ornithine, laminin and fibronectin coating as described above, Matrigel was thawed at 4℃ and diluted 20 × in KnockOut™ DMEM (10,829,018, Gibco), and then added to the same wells. The plates were kept at 37 °C for 2 h, and Matrigel was removed before use.

**POM:** After overnight coating of poly-l-ornithine, the same wells were coated with Matrigel (1:50) at 37 °C for 2 h, and Matrigel was removed before use.

**0.1% PEI Solution:** 50% PEI (P3143, Sigma) was diluted to 10% with sterile water as recommended by FujiFilm Cellular Dynamics, Inc. (https://www.fujifilmcdi.com/icell-motor-neurons-01279-gmnc01279). The 0.1% PEI working solution was freshly made by diluting 10% PEI at a 1: 100 ratio with 1 × borate buffer, which was made by diluting 20 × borate buffer (28,341, Thermo Scientific) with sterile water. The 0.1% PEI was filtered with a 0.22 μM sterile filter (A16534K, Lennox) before use.

### MEA recording and data analyses

MEA recording was performed as described in a recent publication^39^. Briefly, the CytoView 48-well MEA plate (M768-tMEA-48W, AXION) was used to record the electrical signals of MNs. Neuronal activities were recorded for 300 s every other day before medium change, from day 18 to 48. In short, the 48-well MEA plate was docked into the Maestro MEA recording amplifier with a heater to maintain at 37 °C (Axion Biosystems). Signals were sampled at 12.5 kHz, digitized and analyzed using Axion Integrated Studio Navigator (AxIS) 2.5.2 with a 200-Hz high-pass and 3-kHz low-pass filter. An adaptive spike detection threshold was set at 5.5 times of the standard deviation for each electrode with 1-s binning^35^.

The exported spike files (.spk) were batch-processed using Neural Metric Tool (Axion Biosystems). All data reflect well-wide averages from active electrodes, with the number of wells per condition represented by n values. Briefly, an active electrode was defined as having ≥ 5 spikes/min, Mean Firing Rate (Hz) as the total number of spikes divided by the total number of electrodes (16 electrodes per well) over a recording duration (300 s), and weighted Mean Firing Rate (Hz) as the total number of spikes divided by the number of active electrodes. A single electrode burst detector was set to detect the inter-spike interval (ISI) threshold with ≥ 5 spikes in a maximum of 100 ms ISI. The network burst detector was set to 50 spikes with a maximum of 100 ms of ISI, with > 35% of active electrodes involved in bursting. The network burst rate was calculated as the total number of network bursts divided by the recording duration (300 s). The synchrony index was calculated using a Cross-Correlogram Synchrony Window of 20 ms^57^. The processed data were exported as a comma format.csv file. For comparison (i.e., time-course, control-patient pair), the.csv file was uploaded to the Axion Metric Plotting Tool. The summarized.csv file with parameters was exported for statistical analysis. The more detailed description can be found in the Fig. S3.

### Statistical analysis

Statistical analyses were performed with GraphPad Prism version 9.3.1 using unpaired student’s *t* test with a *p < 0.05, **p < 0.01, ***p < 0.001. N represents the total number of cell lines, n is the number of independent experiments or the MEA wells used. Data are presented as the mean ± SEM.

## Electronic supplementary material

Below is the link to the electronic supplementary material.

Supplementary Material 1

Supplementary Material 2

## Data Availability

Data is provided within the manuscript or supplementary information files.
