# Supplementary material for: Polyethyleneimine facilitates the growth and electrophysiological characterization of iPSC-derived motor neurons
Source: Sci Rep. 2024 Oct 30;14:26106. doi: 10.1038/s41598-024-77710-1 (PMC11525838; doi:10.1038/s41598-024-77710-1)
Supplement: Supplementary file 1 — Supplementary Material 1 [file 41598_2024_77710_MOESM1_ESM.docx]

**Supplementary figures and tables**

**Figure S1.**


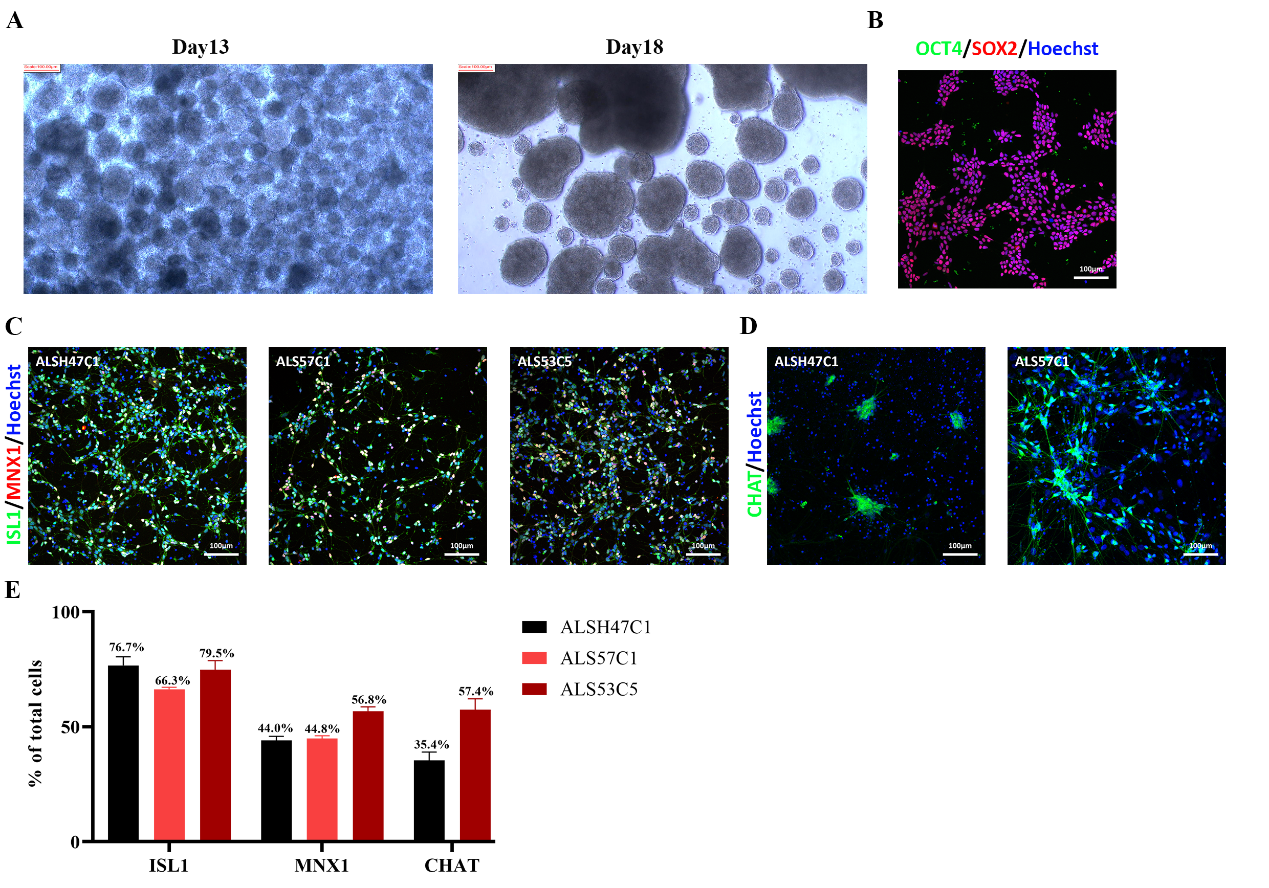


**Figure S1. MN differentiation and characterization following a previously published protocol. (A)** Representative images of suspension culture on day 13 and day 18 of differentiation. **(B)** Representative double staining of OCT4/SOX2, indicating the silence of iPSC pluripotent marker, OCT4 on day 6 of differentiation. **(C)** Representative double staining of MN markers, ISL1/MNX1 from multiple iPSC lines on day 18 of differentiation **(D)** Representative double staining of MN mature marker, CHAT from multiple iPSC lines on day 28 of differentiation. **(E)** The efficiency of ISL^+^ and MNX1^+^ MNs on day 18 and CHAT^+^ mature MNs on day 28. MNP differentiation on day 12. Scale bar 100µM.

**Figure S2.**


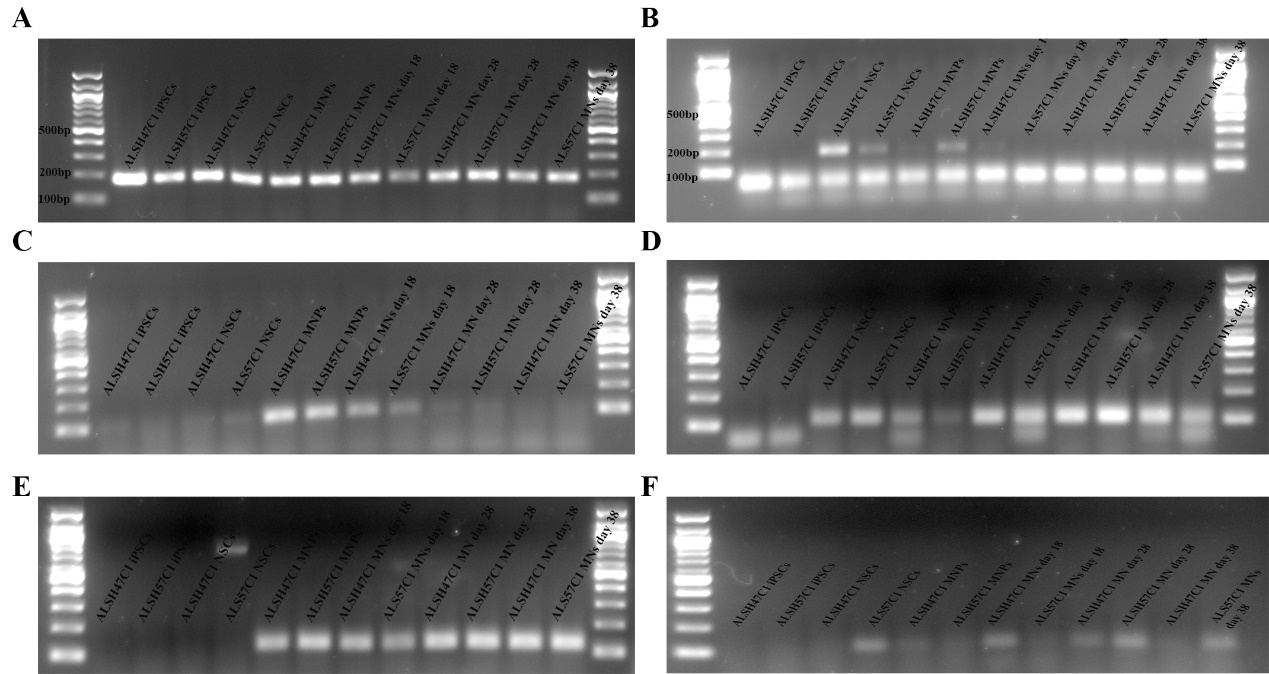


**Figure S2. Full-length gels of the RT-PCR images in Figure 1. (**A: GAPDH; B: PAX6; C: OLIG2; D: ISL1; E: MNX1; F: CHAT.)

**Figure S3.**


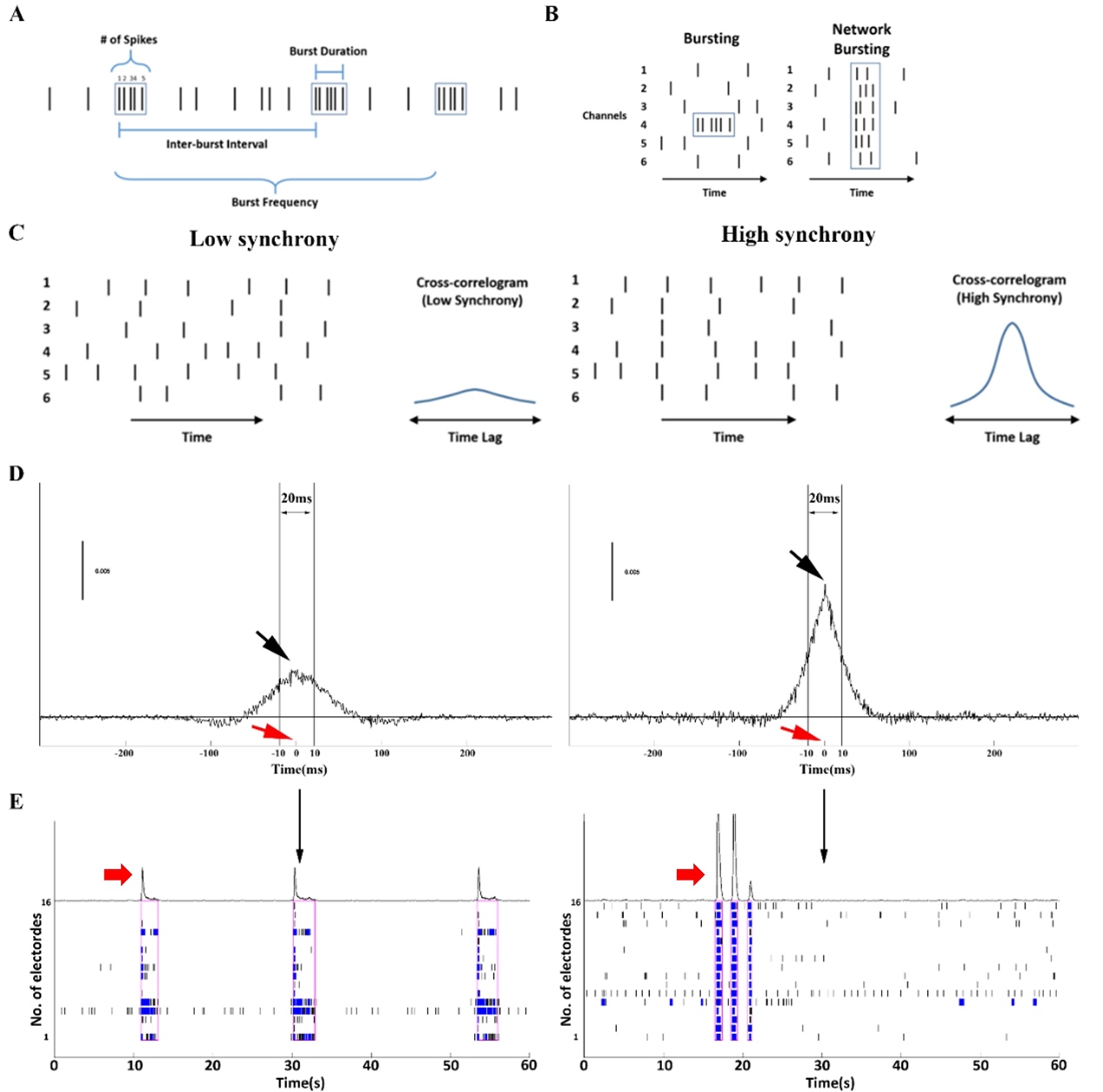
Figure S3. Metrics diagram shows the types of neuronal activity detected by MEA. (A) Each tick represents the time a neuronal AP, or “spike” detected, and each row indicates the electrode (blue boxes). A cluster of spikes on a single electrode is called a burst. The time between two single spikes is defined as Inter-Spike-Interval (ISI). If ≥ 5 spikes are detected from a single electrode in 100ms ISI, the cluster of spikes is considered a burst in this study. (B) A network burst is a coordinated cluster of spiking across multiple electrodes (16 electrodes in each well of a 48-well MEA plate) (blue box in the right panel). If >35% electrodes in a well detect ≥ 50 spikes in 100ms ISI, the coordinated cluster of spikes is considered a network burst in this study. (C) [Schematic diagram](javascript:;) showing low (left) and high (right) synchrony. Synchrony indicates the level of coordinated or simultaneous spiking between electrodes. It is quantified using a cross-correlogram between spiking on pairs of electrodes and then pooling across all pairwise combinations in a well (Halliday et al., 2006). The cross-correlogram assesses the probability of a spike occurring on electrode A at times relative to a spike on electrode B. This probability is summed across all spikes in electrode B to produce the cross-correlogram. For example, if both electrodes always fire together, the cross-correlogram would have a sharp peak at time 0. (D) Neural Metric Tool computes synchrony by first finding the cross-correlation. Phase lags of zero (x-axis on correlogram, red arrow) indicate synchronous spiking between electrodes. When more electrodes have a similar phase relationship, the probability (y-axis height on the correlogram, black arrow) of that phase lag (zero for synchrony) increases. The normalized cross-correlation normalizes the cross-correlation to remove the autocorrelation – each electrode’s cross-correlation with itself. Tall, sharp cross-correlograms indicate high synchrony (right panel). Short, wide cross-correlograms indicate low synchrony (left panel). (E) Raster plot showing low (left) and high (right) neuronal network activities. Data is presented from one patient iPSC-MNs. The ticks indicate single spikes, and the series of blue ticks indicate bursts. The magenta boxes represent that a network burst is formed across the recording well. ﻿Above the raster is a filtered population spike time histogram, the total number of spikes occurring throughout the well at each time (red arrows). A higher peak represents more spikes are detected at that time point, which reflect the synchrony index in some extent.

**Figure S4.**


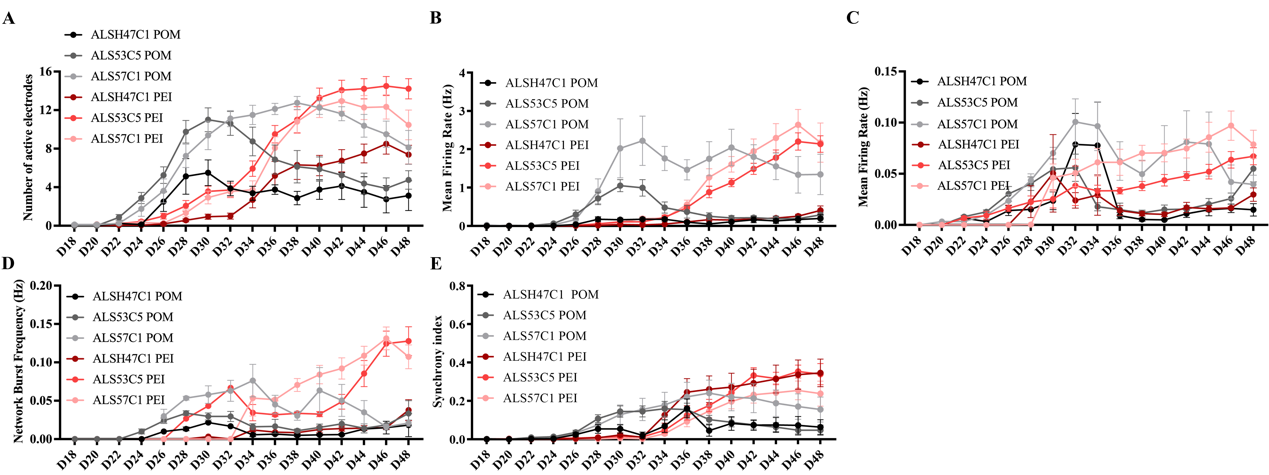


Figure S4. Longitudinal MEA recording of each cell line during D18 to D48 of sMNs plated on POM or PEI. (A) The number of active electrodes, (B) mean firing rate (Hz), (C) burst frequency (Hz) (D) network burst Frequency and (E) synchrony index of each cell line involved in the electrophysiological comparison. The total number of wells of each cell line were: ALSH47C1 POM (dark) n=8, ALS53C5 POM (grey) n=8, and ALS57C1 POM (light grey) n=8, ALSH47C1 PEI (dark red) n=16, ALS53C5 PEI (red) n=14, and ALS57C1 PEI (pink) n=15.

**Reference**

Halliday, D.M., Rosenberg, J.R., Breeze, P., and Conway, B.A. (2006). Neural spike train synchronization indices: definitions, interpretations, and applications. IEEE transactions on bio-medical engineering *53*, 1056-1066.
